# National user fee abolition and health insurance scheme in Burkina Faso: How can they be integrated on the road to universal health coverage without increasing health inequities?

**DOI:** 10.7189/jogh.10.010319

**Published:** 2020-06

**Authors:** Frank Bicaba, Lalique Browne, Kadidiatou Kadio, Alice Bila, Abel Bicaba, Thomas Druetz

**Affiliations:** 1 Société d’Études et de Recherches en Santé Publique (SERSAP), Ouagadougou, Burkina Faso; 2 University of Montreal School of Public Health, Montreal, Canada; 3 Institut de Recherches en Sciences de la Santé (IRSS), Ouagadougou, Burkina Faso; 4 Centre de Recherche en Santé Publique, Montreal, Canada; 5 Center for Applied Malaria Research and Evaluation, Tulane University, New Orleans, Lousiana, USA

In May 2016, the government of Burkina Faso introduced a free health care policy for pregnant women and children under five years of age. This national policy has been effective since in all public health facilities, regardless of their level in the pyramid of care – Burkina Faso counts 1760 primary care facilities, 47 medical centers with surgical capacities, and 13 reference hospitals at the regional or national levels. The health system is complemented by an increasing network of community health workers who provide free services – including test and treatment for certain diseases– to their local communities.

It is noticeable that Burkina Faso is one of the first countries in the Region to overturn the cost recovery policy which imposed direct payment for health care in most sub-Saharan African countries following the Bamako Initiative in 1986. State expenses in health care have gradually increased in the last years; totalizing US$330 million in 2018, ie, 10% of the total State budget. In 2016, the year of the introduction of the free health care policy, the government health expenditures represented 40% of the total expenditures in health, compared to 28% the previous year. On the other hand, the proportion of health expenditures directly paid by patients (out-of-pocket expenses) decreased from 36% to 31% during the same period.

Scientific evidence of the positive impact of free health care has been gathered in Burkina Faso as well as elsewhere in sub-Saharan Africa. Exempting children under five years of age from direct payment increases their use of primary health services, increases the propensity of visiting a health center in the event of an episode of illness (at the expense of self-medication and traditional treatments), and increases the promptness of such use [1]. For mothers and pregnant women, free health care increases pre- and post-natal consultations as well as assisted childbirth and caesarean section rates [2]. A growing number of studies also suggest a positive impact of free health care on indicators of maternal, newborn and child mortality and morbidity [3]. Finally, it is essential to highlight the evidence that free health care in Burkina Faso contributes to reducing health inequalities [4].

Despite these results and the support of the country's leaders, this policy is currently facing a major obstacle: the issue of sustainable financing. Indeed, the Burkinabe State reimburses the costs associated with free health care (consultations, medicines, treatments) to health centers, which serve as third-party payers. In 2018, the total sum to be repaid amounted to CFAF 32 billion ( ∼ US$54 million, ie, 16.3% of the government health budget) directly attributable to the State's regular budget, which wished to free itself from external aid in the implementation of the policy. Approximately 55% of this amount could not be reimbursed in 2018, and constituted a debt in 2019. In this context of high pressure, careful consideration is being given to releasing budgetary resources to ensure the continuity of the free health care policy (and its extension to family planning) without depending on external aid.

One of the solutions being considered is to incorporate the free health care policy into the Universal Health Insurance (UHI) scheme. Introduced last year, this scheme is in line with the Universal Health Coverage and plans to improve access to health care for other segments of the population through contribution to an insurance scheme. As a first step, participation in the UHI scheme would be mandatory for all public service employees and voluntary for the rest of the population. For all members, direct payments to health centers would be considerably reduced and would be limited to co-payments. However, due to the vulnerability of these target populations, health care would continue to be completely free of charge for children under 5 years of age and for maternal health care. The long-term ambition is for the new UHI national fund to generate surpluses that would partly contribute to sustaining the free health care policy, which would remain effective. Burkina Faso would also join other countries that have combined free health care with insurance schemes, such as Morocco and Ghana.

The mechanisms for linking the two interventions are not yet established and continue to be the subject of discussions between stakeholders; however, preparatory interviews conducted as part of another study revealed that the consensus option could be to make free health care conditional to UHI membership. This approach implies that in order to continue to benefit from free health care, the target population would first have to join the UHI National Fund and pay their contributions. The addition of this eligibility criterion to free health care would encourage households to join the UHI and increase the population penetration rate, which is one of the main implementation challenges in new universal insurance schemes. On the other hand, there is a significant risk that contribution fees will represent an overly burdensome financial barrier for a majority of households, resulting in the return of direct payments for maternal health care and for children under 5 years of age. Almost inevitably, such a mechanism would encourage the restoration of health inequalities, since households with the lowest incomes would be less likely to join the UHI, which would disproportionately reduce their access to health care. After years of interventions that have significantly reduced health inequalities in the country, such a scenario would likely reverse this trend.

Beyond this social justice issue, the return of direct payments may also have an impact on a range of health indicators. Indeed, we have observed such a phenomenon in the Kaya district, where a free health care pilot project was introduced in 2010, then interrupted in January 2014, before finally being reintroduced a few months later. Our analyses showed that visits to health centers decreased immediately and significantly when free health care was withdrawn [5]. This natural experiment showed that demand for health care remained very elastic to the cost of consultations, particularly in rural areas. The gains obtained after years of free health care are precarious and could be erased quickly if a form of direct payment was reintroduced.

However, there are other options for linking free health care to an insurance plan while limiting the risk of increasing health inequities. Several of these options have been tested in other contexts and could contribute to the sustainability of the free health care policy while exempting the most vulnerable populations from the obligation to contribute to the UHI. The first of these options would be to exempt indigents, who generally include those who have no income and are unable to provide for themselves, from paying contributions. There have been several programs aiming to exempt indigents from direct payments in health centers, including in Burkina Faso. However, mechanisms to identify indigents have faced numerous implementation issues and have often led to the exclusion of indigents and to the creation of conflicts inside the community [6]. These programs are also insufficient to ensure equitable access to health care [7]. The second option aims to exempt workers in the informal sector from paying contributions, a measure that has proven effective elsewhere in increasing coverage among the most vulnerable populations [8]. However, in Burkina Faso, it is estimated that the formal economy represents only 20% of the urban labour force, and about 1% of the rural labour force. Therefore, such an exemption would greatly limit the contributions of the UHI National Fund. Lastly, rather than exempting to categorize individuals, the linkage mechanism could be based on the exemption of geographical regions, reflecting the phenomenon of poverty concentration in particular areas. This mechanism is particularly recommended in countries with a high prevalence of poverty [9]. In the Burkinabe context, a variation to consider would be to continue to provide free health care to mothers and children under 5 years of age in primary health centers located in rural areas, without requiring proof of UHI contribution.

**Figure:**
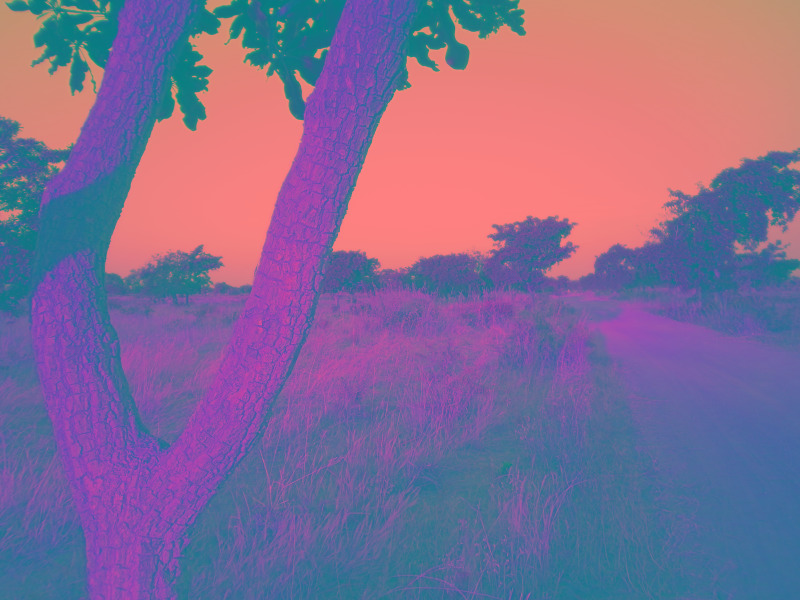
Photo: Rural communities in Burkina Faso need to be prioritized in order to improve universal health coverage (from the collection of Thomas Druetz, used with permission).

The challenges raised by sustainability of the free health care policy in Burkina Faso deserve particular attention. The linking of free health care with the UHI National Fund, both of which are measures aiming to improve universal health coverage, raises issues of efficiency, sustainability and social justice. While many scientific studies focus on a single ideal means to achieve the objective of UHC, it is increasingly apparent that in some contexts, a combination of several interventions is necessary. From this perspective, it is urgent to redirect research towards mechanisms for linking these interventions and to rigorously weigh the potential risks and benefits of the various options, with the fundamental issue of health equity as a decision-making prism. Finally, the incorporation of free health care into a universal mandatory insurance mechanism is one avenue among others. The introduction of a “nutritional tax” on sweetened drinks or on high-fat products could contribute to the sustainable financing of a free health care policy, the positive and equitable impact of which has been demonstrated many times[10].
